# Popliteal Aneurism

**Published:** 1830-07-01

**Authors:** 


### XLVI.

### WESTMINSTER HOSPITAL.

There have been few capital operations or cases of importance here since our last report. Two cases of popliteal aneurism, for which the operation has been performed, are sufficiently interesting, however, to be worthy of more particular notice.

### POPLITEAL ANEURISM.

CASE 1. *Operation — cure.* William Cooper, æt. 26, admitted, March 24th, under the care of Mr. Guthrie. He is a farmer's labourer, residing generally some distance from London, tolerably strong and muscular in appearance, and has commonly enjoyed very good health until within the last two months, when he suddenly experienced, while walking, a sensation like that of cramp in the left ham. This feeling occasionally returned, but did not produce such inconvenience as to prevent him following his usual occupations; about four weeks since, the pain and symptoms generally became more serious, and he applied for medical advice. The gentleman whom he consulted informed him he had an aneurism, and recommended him to procure addmission into some hospital. He rolled his leg for some time with a bandage, from which he suffered an increase of pain and inconvenience.

*Present State.* The thorax is well developed. Percussion elicits an audible sound all over the chest, which expands fully at each inspiration. Respiratory murmur natural, and lungs apparently quite healthy. The action of the heart is regular—the systole of the auricles and ventricles is perfectly distinct, both in the left intercostal space and at the base of the sternum. The force of the heart is much increased and its action is perceptible over the whole of the anterior part of the chest and in the axillary regions.

The patient is in other respects perfectly healthy. A tumour exists in the left popliteal space, making a considerable prominence at the point of flexion; this may be made to disappear by steady pressure, and its pulsation is synchronous with that of the artery, and equally distinct in all parts of the tumour. Pulsation may be entirely stopped by pressure on the femoral artery or the groin. The patient complains of a dull pain in the part extending down the calf. The whole of this leg is larger than the other, and slightly œdematous. Ordered low diet, beef tea, and one egg daily—to be kept quiet in bed.

March 28th. His bowels have been freely acted upon by house-medicine, but he has a hard, full pulse, 92 in the minute, and he presents some symptoms of inflammatory action; he was bled yesterday to ℥viij. The blood abstracted is slightly buffed and cupped, on which account the operation has been postponed.

30th. The same symptoms are still observable, though less strongly marked. V.S. ad ℥xiv. The appearance of the blood is nearly natural.

April 4th. Much the same—pulse 72, soft and full. V.S. ad ℥xij.

5th. The blood drawn yesterday is slightly buffed, but less than at first. The tumour is gradually increasing in size, and the dull pain continues in his leg, increasing towards night—tongue pale, bowels open. Mr. Guthrie directed that the temperature of both legs should be ascertained. At 10, a.m. the temperature of the right leg was 92, and the left the same; at 10, p.m. that of both was 96°.

10th. At one o'clock, a.m. The operation of tying the femoral artery in the upper third of the thigh was performed.

*Vespere.* He complains of shooting pains in the knee and calf of the leg; pulse full. Hot flannels are applied to the limb and bottles of hot water to the foot. Temperature of right leg 86°—left 82°. 10, p.m. The patient has slept a little, and the pain has abated considerably. Pulse 80, rather full—temperature of the right leg 93°, left 91°.

April 11th, 8, a.m. He has passed a comfortable night, but he complains to Mr. Guthrie this morning of pain where the incision was made. The leg is apparently more swollen, and the veins slightly distended—no pulsation in the tumour, and the temperature of both legs is 92. Pulse 100—tongue furred and whitish, skin hot, and he complains of pain in the head. Bowels have not been opened since the operation.

℞. Potassæ Carb., ℈j.

Sacci Limonis, ʒiij.

Aquæ.. ..... ℥x.

Sodæ Sulph .. ʒj. M. ft. haust.

4tis horis. sumend.

6, p.m. Bowels have not acted, but pain in the head is relieved; pulse 100, and not very easily compressed: temperature of the right leg 98—left 96.

℞. Hyd. Submur. gr. iv.

Ext. Col. c... gr. vj. Ft. pil ij. st. sumend. Mist. salin. contin. 2dis horis donec alvus respondet.

12th. 8, a.m. Two doses of castor oil were also administered last night; the bowels have, however, only been imperfectly acted upon. He has slept tolerably well; skin is less hot; pulse the same. He complains of slight shooting pains in the inner side of the thigh; temperature of both legs 94. V.S. ad ℥xvj. Haus. Aperiens statim.

10, a.m. He has had three evacuations, and the pain in his head is relieved. He has slight pain in the course of the femoral vein; pulse 110; his countenance is less flushed, but he is irritable. The blood abstracted is slightly buffed but not cupped; he says he feels low and weak.

Mist. Salin. c. Liq. Opii Sed. 4tis horis. ♏ iij. pro dosi.

6, p.m. His countenance is more cheerful, and he appears altogether better.

10th. He has suffered from slight pains in the abdomen; the limb is quite easy. Ordered liq. opii sed. ♏xv. st. and to be repeated at 12 o'clock, if necessary.

13th, 8, a.m. The draught was repeated during the night, and after that he slept well, and is now free from pain, but occasionally he experiences shooting pains in the course of the femoral vein; just below the incision there appears a slight blush of redness with fulness, and tenderness on pressure. Temperature of both legs 93°. Pulse 98 and soft; tongue moist and less furred; the diseased leg is not so much swollen, and Mr. Guthrie thinks the tumour has diminished in size since the operation. The strips of adhesive plaister were loosened, and the patient ordered to repeat the aperient draught. Twelve leeches to be applied to the course of the femoral vein, and a bread and water poultice afterwards.

4, p.m. Mr. G. has again seen him: the leeches have bled freely, and he is slightly relieved; in other respects much the same; pulse 110; to be bled at nine o'clock.

10, p.m. Pulse 120, hard and full—V.S. ad ℥xij.—this bleeding has afforded him much relief. Cont. Liq. Opii Sed.

14th, 8, a.m. He has passed a good night, and this morning feels but little pain in the thigh. The blood is buffed and cupped—pulse 110, soft—skin cool—tongue white—bowels freely open—temperature of both legs 92. Rep. med.

10, a.m. Mr. Guthrie has removed the strips of adhesive plaister—the wound appeared healing—a good deal of healthy matter was discharged on pressure, and the wound dressed—there is slight tenderness of the thigh, but the patient suffers very little pain.

10, p.m. The thigh is much easier since the morning. Cap. liq. opii sed. ♏iv.

15th. The patient slept well last night; there is some degree of tenderness on pressure at the lower part of the abdomen, and he complains of inability to void his urine. No pain in the thigh, and great discharge from the wound; skin cool and moist; pulse 82, soft; tongue clean; bowels open.

12, a.m. He has emptied his bladder, and feels now much relieved; a roller, with padding in the course of the femoral vessels, has been applied to prevent the lodgment of matter.

16th. Mr. Guthrie dressed the wound this morning—the patient is going on well—pulse is soft, and he experiences no pain in any part.

April 17th. Dressed by Mr. G. this morning, who observed that the tumour was evidently much diminished, and the veins are less distended.

27th. The patient has continued gradually improving—the wound being dressed every day and the bowels regulated by small doses of sulphate of magnesia. This morning both ligatures came away. Some luxuriant granulations were checked by the application of lunar caustic which produced swelling and tenderness—these were speedily relieved by poulticing.

May 10th. Since the last report the patient's general health has progressively improved—a slight purulent discharge existed for several days, but it has now entirely ceased, and the patient may be pronounced cured.

CASE 2.—*Operation performed—cure.* Thomas M'Caughin, æt. 33, admitted May 14th, with popliteal aneurism in the left leg. He was brought up as a gardener, and has generally enjoyed good health. On the 2d of April last while riding at a good pace, his knee was crushed between another saddle and his own—he says the holster of his comrade's struck him in the ham. The whole limb from the knee downwards swelled and became very painful—unattended by any discoloration of the integuments, and he continued his usual occupation the succeeding four days. The day after the receipt of the injury, whilst walking, he first perceived the pulsation in the left ham, which continued to increase till he came to London, and was received into the hospital. The patient appears in sound health—his chest is well formed, broad, and capacious—action of the heart regular. Pulse 64—bowels open—tongue clean—appetite unimpaired. Temperature of the affected leg 88. Ordered to bed and to have low diet.

May 15th. The man was placed upon the table and Mr. Guthrie proceeded to the operation—having first traced the course of the artery he made his incision in that direction above the sartorius. The artery was readily found and tied, with scarcely any displacement of the parts—a small artery was divided before he came to the femoral, but was not very troublesome. Pulsation stopped in the tumour immediately the artery was tied. A single thread of dentist's silk was used. Mr. G. observed after the operation to the pupils that this patient would probably recover in less time than the one on which he had operated some time previous—that in the latter having cut a considerable branch close to the trunk, he had thought it right to place two ligatures upon the artery, in order to guard against secondary hemorrhage which it might have produced—hence in all probability, the cause of the discharge and protracted union—the intermediate portion having to slough away—he concluded by some observations on the mode of tying the artery, and the necessity of disturbing the parts as little as possible.

5, p. m. Mr. G. has seen him—the tumour appears already diminished—the veins are slightly distended—temperature of the limb 88. The limb is placed on pillows in the half flexed position, and flannel wrapped round it. He complains of sickness with constant pain around the thigh and ankle joints—no numbness of the leg is experienced—Cap. Liq. Opii. Sed. ℳxv.

16th. 8 a. m. He has passed a comfortable night—no febrile excitement—thigh and leg easy—pulse 68—bowels not opened since the operation—tongue moist—skin natural—countenance cheerful.

P. m. Feels rather sick, bowels have not been relieved since the operation—complains also of inability to void his urine—no pain, but a sensation of fulness in the pudic region—tongue moist and covered with a whitish fur—pulse 74—no pain in the limb—temperature 92°.

17th. 8, a.m. He has passed a comfortable night—bowels have been freely opened, and he complains of no sickness—pain, or sensation of numbness—

temperature of the left leg 93°, of the right 92°.

18th. He has passed a good night—experiences no pain in either thigh or leg—bowels open, tongue moist—skin cool—pulse 74. The temperature of the affected limb is 92°. He takes no medicine.

19th. The wound has been dressed for the first time, and was found nearly united by the first intention.

June 13th. He has continued without an unfavourable symptom, and the ligature came away to day—being the 31st after the operation.

The advantages arising from allowing the aneurisms to continue for at least six weeks until the collateral branches had enlarged sufficiently to maintain the life of the limb have been here very apparent. The maintenance of the circulation never having been doubtful in either instance — the tumours rapidly but steadily diminished so as to leave no doubt of the cure being complete.

The operations were both performed in the upper third of the thigh, so that the ligatures were applied immediately by the side of the upper edge of the sartorius, scarcely displacing that muscle in the slightest degree, a very small portion of which was visible. In the 1st case, an artery running to the triceps muscle was divided and bled so freely as to fill the wound with blood and to require compression on the artery above to suppress the hemorrhage, for which reason Mr. Guthrie said he applied two ligatures, the one above and the other below it in the same manner as he would have done if the artery had been wounded.

---

